# Unexpectedly High False-Positive Rates for *Haemophilus influenzae* Using a Meningoencephalitis Syndromic PCR Panel in Two Tertiary Centers

**DOI:** 10.3389/fcimb.2021.639658

**Published:** 2021-03-08

**Authors:** Marie-Céline Zanella, Abdessalam Cherkaoui, Vladimira Hinic, Gesuele Renzi, Daniel Goldenberger, Adrian Egli, Jacques Schrenzel

**Affiliations:** ^1^Laboratory of Bacteriology, Division of Laboratory Medicine and Division of Infectious Diseases, University of Geneva Hospitals, Geneva, Switzerland; ^2^Division of Infectious Diseases, University of Geneva Hospitals, Geneva, Switzerland; ^3^Division of Clinical Bacteriology and Mycology, University Hospital Basel, Basel, Switzerland; ^4^Applied Microbiology Research, Department of Biomedicine, University of Basel, Basel, Switzerland; ^5^University of Geneva Medical School, Geneva, Switzerland

**Keywords:** *Haemophilus influenzae*, false-positive, panel, diagnostics, meningitis, central nervous system infection

## Abstract

False-positive results in the diagnostic of meningitis and encephalitis pose important challenges. This study aimed to determine false-positive rates for *Haemophilus influenzae* in cerebrospinal fluids evaluated by the BioFire FilmArray^®^ Meningitis/Encephalitis Panel. We conducted a retrospective study of all *H. influenzae*-positive FilmArray^®^. Meningitis/Encephalitis Panel results from June 2016 to October 2019 in two Swiss university hospitals. Cases were classified as true positive, likely true-positive, and likely false-positive results according to cerebrospinal fluid culture, *H. influenzae*-specific quantitative real-time PCR (qPCR), and Gram staining, as well as culture of other materials. We performed 3,082 panels corresponding to 2,895 patients: 0.6% of the samples (18/3,082) were positive for *H. influenzae*. Culture and *H. influenzae*-specific qPCR were performed on 17/18 (94.4%) and 3/18 (16.7%) cerebrospinal fluid samples, respectively; qPCR was negative in all cases. Among 17 samples sent for culture, 10 concerned patients were not treated with antibiotics prior to lumbar puncture. Only 1/17 revealed growth of *H. influenzae* and was classified as a true positive. We further classified 3/18 (16.7%) cases with the identification of Gram-negative rods in the cerebrospinal fluid or positive blood cultures for *H. influenzae* as likely true-positive and 14/18 (77.8%) cases as likely false-positive. Diagnostic results should always be interpreted together with the clinical presentation, cerebrospinal fluid analysis, and other available microbiological results. All *H. influenzae*-positive results should be viewed with special caution and a *H. influenzae-*specific qPCR should be systematically considered.

## Introduction

Central nervous system (CNS) infections are commonly caused by diverse pathogens, mostly viruses and bacteria, and molecular multiplex panels have been developed to simultaneously diagnose multiple pathogens in cerebrospinal fluid (CSF) samples. These molecular rapid diagnostic tests are increasingly used in routine diagnostic laboratories for the diagnosis of CNS infections, including meningitis and meningoencephalitis, with the advantages of being easy to use and having short turn-around times. Nevertheless, these syndromic panels lack extensive analytical and clinical validation and concerns have been raised regarding their suboptimal performances (Vetter et al., 2020). Several cases of false-positive results for some targeted pathogens, more specifically for *Haemophilus influenzae*, have been reported with the BioFire FilmArray^®^ Meningitis/Encephalitis (ME) Panel (bioMérieux, Lyon, France) (Naccache et al., 2018; Boudet et al., 2019). False-positive results could lead to additional investigations and inappropriate antibiotic prescriptions.

*H. influenzae* is associated with upper and lower respiratory tract infections, but also with invasive infections including meningitis (Agrawal and Murphy, 2011). The broad use of the *Haemophilus influenzae* type b (Hib) conjugate vaccine led to a dramatic reduction of Hib infections in various countries including Switzerland, where the incidence in 2019 was 1.43 of confirmed cases per 100,000 population (Federal Office of Public Health, Division of Communicable Diseases, [[NoYear]]; Agrawal and Murphy, 2011). In Switzerland, the reported vaccination coverage for children is 89% for the 2014–2016 period (Federal Office of Public Health, Division of Communicable Diseases, 2018).

After implementation of the FilmArray^®^ ME Panel, the bacteriology laboratories of Geneva and Basel university hospitals witnessed an unexpectedly high number of positive cases of *H. influenzae*, raising suspicion of false-positive cases. In this context, the aim of this retrospective study was to analyze all reports of *H. influenzae* and to determine the proportion of false-positive cases on all CSF samples analyzed in these two laboratories starting from the introduction of the FilmArray^®^ ME Panel.

## Materials and Methods

### Ethics Statement

The study was approved by the Geneva and Basel cantonal ethics commissions (Geneva #2020-00215; Basel #2020-01724).

### Clinical Specimens

We included all samples from pediatric and adult patients for whom a FilmArray^®^ ME Panel PCR (bioMérieux, Lyon, France) was ordered for CSF microbiology testing at the bacteriology laboratories of Geneva and Basel university hospitals from 1 June 2016 (Geneva) and 17 May 2016 (Basel) to 31 December 2019 and reported to be positive for *H. influenzae*. Multiple specimens from single patients were not excluded.

### Routine, FilmArray^®^ ME Panel, and Confirmatory Testing

Both laboratories are accredited (ISO/IEC17025) and regularly participate in external quality control surveys. In both hospitals, all patients with suspected CNS infection benefit from clinical evaluation, prompt administration of antimicrobials, cerebral radiological exams when needed, and lumbar puncture for CSF analysis, according to published guidelines (van de Beek et al., 2016). When sufficient CSF sample volume is available, the following analyses are performed: CSF cellular count and chemistry, and direct examination with Gram stain, culture, and the FilmArray^®^ ME Panel. Further microbiological investigations can be performed according to the clinical presentation and suspected pathogen, local epidemiology, in accordance with published guidelines (van de Beek et al., 2016). *H. influenzae* positive results with the FilmArray^®^ ME panel are confirmed with a specific quantitative real-time PCR (qPCR) as previously reported (Price et al., 2015) when enough CSF sample is available.

The FilmArray^®^ ME Panel was performed on CSF samples in accordance with the manufacturer’s instructions, including laboratory precautions and good microbiological practice to prevent contamination. Briefly, 200 µl of CSF specimen is subjected to panel testing. The FilmArray^®^ ME Panel test consists of automated nucleic acid extraction, reverse transcription, two multiplex nucleic acid PCR amplifications, and detection using DNA melting temperature analysis of 14 of the most common bacterial, viral, and fungal pathogens associated with CNS infections (seven viruses, six bacteria and *Cryptococcus neformans/gatii*) (Leber et al., 2016); results are available within approximately 1 h.

### Data Collection

Chart review was conducted to collect the characteristics of the included patients and data regarding their clinical management. During clinical management, the diagnosis of CNS infection was based on the integration of the pre-test probability, clinical presentation, radiological examinations and CSF analysis results. Patients received antimicrobial treatment according to published guidelines (van de Beek et al., 2016). A review of laboratory data was conducted to collect CSF cell counts, as well as protein and chemistry values of the included CSF samples. Results of culture and other molecular assays were obtained for the included CSF samples and additional clinical samples collected within 48 h around the time of CSF sampling. Medical charts were also reviewed for adverse events attributed to antibiotic treatment and *Clostridioides difficile* infection during a maximum of 2 months in-hospital follow-up.

### Data Analysis

Positive results for *H. influenzae* with the FilmArray^®^ ME Panel and further laboratory tests were classified as follows:

- *True-positive result:* confirmed by CSF culture and/or positive *H. influenzae-*specific qPCR on the same CSF sample.
*- Likely true-positive result:* not confirmed by CSF culture or *H. influenzae-* specific qPCR when performed. CSF cellularity, chemistry analysis, CSF Gram stain results and the culture results of other clinical samples (*e.g.* blood cultures) suggest that the result was likely to be a true positive.
- *Likely false-positive result*: not confirmed by CSF culture or *H. influenzae-*specific qPCR when performed. CSF cellularity, chemistry analysis, CSF Gram stain results and the culture results of other clinical samples (*e.g.* blood cultures) suggest that the result was likely to be a false-positive.

## Results

We performed a total of 3,082 FilmArray^®^ ME Panels corresponding to 2,895 patients (2,252 adult; 643 pediatric patients). Results for *H. influenzae* were negative in 99.4% (3,064/3,082; 95% CI 99.1–99.6%) cases and positive in 0.6% (18/3082; 95% CI 0.4–0.9%) CSF samples, corresponding to 12 adult and six pediatric patients. Clinical characteristics and laboratory data of the 18 cases with positive *H. influenzae* FilmArray^®^ ME Panel results are detailed in **Table 1**. Among the 18 cases, five cases (patients #1, #2, #3, #5, and #6, **Table 1**) and 13 cases had CSF specimens collected and analyzed in Basel and Geneva University Hospital Laboratory, respectively.

**Table 1:** Case classification, patient characteristics and laboratory data of patients with positive *H. influenzae* BioFire FilmArray^®^ ME Panel results (n = 18).

Patient number	Case classification*	Gender	Age (years)	Antibiotics <48 h before LP	CSF and blood chemistry					CSF microbiological testing				Other suspected infection sites	Blood culture results**	Other clinical sample culture results
					CSF WBC (M/L)	CSF PMN (%)	CSF totalproteins (g/L)	CSF glucose (mmol/L)	Blood glucose (mmol/l)	CSF Gram stain	CSF culture	Biofire FilmArray^®^ ME Panel result	Specific *H. influenzae* qPCR			
1	TP	M	5	NA	NA	NA	NA	NA	NA	Gram-neg rods	*H. influenzae*	*H.influenzae*	ND	No	neg (1/1)	
2	Likely TP	M	1	No	NA	NA	NA	NA	NA	Gram-neg rods	neg	*H.influenzae*	ND	Otitis media	*H. influenzae* (1/1)	Ear-swab, *H. influenzase*
3	Likely TP	M	21	Yes	90	65	0.81	3.5	ND	Gram-neg rods	neg	*H.influenzae*	ND	No	neg (4/4)	
4	Likely TP	F	2	No	4031	78	0.77	3.9	7.6	Absence	neg	*H.influenzae*	ND	Otitis media	*H. influenzae* (1/1)	
5	Likely FP	M	79	No	5	0	1.22	9.1	ND	Absence	neg	*H.influenzae*	ND	No	neg (4/4)	
6	Likely FP	M	11	No	NA	NA	0.99	2	ND	Gram-pos cocci	*S.aureus*	*H.influenzae*	ND	Ventricular shunt infection	neg (1/1)	
7	Likely FP	M	12	Yes	3	58	0.85	2.8	3.7	Absence	neg	*H.influenzae*	neg	No	ND	
8	Likely FP	F	34	Yes	1	54	0.45	3.9	6.3	Absence	neg	*H.influenzae*	ND	No	neg (4/4)	
9	Likely FP	F	1 month	No	7	20	0.65	2.5	ND	Absence	neg	*H.influenzae*	neg	No	*Actinomyces* sp.^†^ (1/1)	
10	Likely FP	F	26	Yes	230	10	0.71	3.4	ND	Absence	neg	*H. influenzae*, enterovirus	neg	No	neg (4/4)	
11	Likely FP	M	49	Yes	2	1	1.03	4.3	5.9	ND	ND	*H.influenzae*	ND	No	neg (4/4)	
12	Likely FP	F	22	Yes	<1	ND	0.28	3.8	5.7	Absence	neg	*H.influenzae*	ND	No	ND	
13	Likely FP	M	82	No	4	18	0.86	3.7	5.4	Absence	neg	*H.influenzae*	ND	No	neg (2/2)	
14	Likely FP	M	77	No	3623	98	3.89	7.8	13.2	Gram-neg rods	*E. coli* K1	*H. influenzae*, *E. coli K1*	ND	No	neg (6/6)	
15	Likely FP	F	46	No	<3	0	0.29	ND	ND	Absence	neg	*H.influenzae*	ND	No	ND	
16	Likely FP	F	73	Yes	2	9	0.31	5.4	5.1	Absence	neg	*H.influenzae*	ND	No	ND	
17	Likely FP	M	38	No	14	12	0.66	3.2	ND	Absence	neg	*H.influenzae*	ND	No	ND	
18	Likely FP	F	43	No	9	5	0.39	3.4	5.1	Absence	neg	*H.influenzae*	ND	No	ND	

*See Materials and Methods section for case classification definitions.

** Number of positive or negative blood cultures bottles among total blood cultures sampled within 48 h around the time of the lumbar puncture.

^†^Considered as a contaminant.

FP, false-positive; TP, true-positive; M, male; F, female; LP, lumbar puncture; CSF, cerebrospinal fluid; qPCR, quantitative real-time PCR, WBC, white blood cell count; Lympho, lymphocytes; PMN, polymorphonuclear; H. infl., H. influenzae; S. aureus, Staphylococcus aureus; E. coli, Escherichia coli; neg, negative; pos, positive; ND, not done; NA, not available.

Culture and *H. influenzae*-specific qPCR were performed on 17/18 (94.4%) and 3/18 (16.7%) CSF samples, respectively. *H. influenzae*-specific qPCR assay was performed in three samples only due to the limited CSF sample volume available for further testing; qPCR was negative in three cases. Among 17 samples sent for culture, 10 concerned patients were not treated with antibiotics prior to lumbar puncture and only one revealed the growth of *H. influenzae*. This case (patient #1, **Table 1**) was classified as a true positive. We classified three (16.7%) cases as likely true-positive, with the identification of Gram-negative rods on CSF gram stain in two cases (patients #2 and #3, **Table 1**) and positive blood cultures for *H. influenzae* in two cases (patients #2 and #4, **Table 1**). Of note, patients #2 and #4 were diagnosed with *H. influenzae* otitis media. The remaining 14 (77.8%) cases were classified as likely false-positive cases (**Table 1**).

CSF white blood cell count was determined in 13 samples (median 7 M/L [range 1–4,031 M/L]). Among patients with an elevated CSF white blood cell count, two cases were classified as likely true-positive *H. influenzae* results (patients #3 and #4, **Table 1**), and two as likely false-positive with another retained cause of CNS infection (patient #10 with enterovirus-associated meningitis and patient #14 with *Escherichia coli*-associated meningitis, **Tables 1** and **2**).

**Table 2:** Alternative diagnosis, clinical management, and reported adverse effects of antibiotic treatment based on the retrospective review of medical charts of patients with positive *H. influenzae* BioFire FilmArray^®^ ME Panel results (n = 18).

Patient number	Case classification*	Alternative diagnosis	Final retained alternative diagnosis	Treated as bacterial meningitis with full course of antibiotics	Reported CDI or adverse event associated with antibiotics
1	TP	No	None	Yes	NA
2	Likely TP	No	None	Yes	NA
3	Likely TP	No	None	Yes	No
4	Likely TP	No	None	Yes	No
5	Likely FP	Yes	Encephalopathy/encephalitis of unknown etiology	Yes	No
6	Likely FP	Yes	*S. aureus*-associated ventricular shunt infection	Yes	NA
7	Likely FP	Yes	Influenza A-associated meningo-encephalitis	Yes	No
8	Likely FP	Yes	Epilepsy crisis associated with benzodiazepine withdrawal	No	No
9	Likely FP	Yes	Fever of unknown origin	No	No
10	Likely FP	Yes	Enterovirus-associated meningitis	No	No
11	Likely FP	Yes	Epilepsy crisis associated with cerebral toxoplasmosis and pachymeningitis of unknown origin	No	No
12	Likely FP	Yes	Fever and headache (suspicion of upper respiratory tract infection)	No	No
13	Likely FP	Yes	Epilepsy and delirium after cranial trauma	No	No
14	Likely FP	Yes	*E. coli* K1-associated meningitis	Yes	No
15	Likely FP	Yes	Delirium of unknown origin in a patient with HIV infection	No	No
16	Likely FP	No	None	Yes	No
17	Likely FP	Yes	Chronic pachymeningitis of unknown origin	Yes	No
18	Likely FP	Yes	Multiple sclerosis	No	No

*See Material and Methods section for case classification definitions.

FP, false positive; TP, true positive; NA, not available. CDI, Clostridioides difficile infection. S. aureus, Staphylococcus aureus. E. coli, Escherichia coli.

Regarding the clinical management, the review of medical charts identified that an alternative diagnosis was made for 13/18 (72.2%) patients (**Table 2**). All cases classified as likely true- positive were treated for *H. influenzae* meningitis (**Table 2**). Among cases classified as likely false-positive, only four were treated with antibiotics for bacterial meningitis (**Table 2**). No adverse event associated with antibiotic treatment or *Clostridioides difficile* infection was reported (**Table 2**).

Details of the amplification curve results of the initial and repeated FilmArray^®^ ME Panels when performed are provided in **Table S1**. Only 1/18 (5.5%) samples revealed positive amplification curves on the two targets designed to detect *H. influenzae* (patient #4, **Table S1**). Among three patients for whom the FilmArray^®^ ME Panel was repeated on the same CSF sample, results were positive in two patients and classified as likely true-positive cases (patients #2 and #4, **Table S1**).

## Discussion

In this two-center retrospective study conducted over a period of 3 years, the proportion of CSF samples with *H. influenzae* FilmArray^®^ ME Panel positive results was 0.6% (95% CI 0.4–0.9%). A review of the 18 *H. influenzae* positive results revealed that only one case (5.5%) could be considered as a true positive. Regarding *H. influenzae* culture-positive results from other clinical samples, Gram stain and CSF analysis, 16.7% of cases were classified as likely true-positive cases. At the time of clinical management, these patients have been treated accordingly. For a high proportion of cases (77.8%), CSF analysis and microbiological results suggested that *H. influenzae* FilmArray^®^ ME positive results were likely false-positive. Among these, the review of medical charts suggested that *H. influenzae*-associated meningitis was finally retained in a few patients: an alternative diagnosis was made in 92.9% of patients and only 28.6% received a full course of antibiotics for bacterial meningitis. The fact that only a minority received antibiotics highlights that the result was not in concordance with the clinical picture and course of the patient. The impact of the false-positive results was therefore limited since panel results were interpreted together with clinical manifestations, CSF analysis, and other microbiological results. Importantly however, neither the validation studies performed before panel implementation nor the regular EQC is designed to detect that rate of false-positive results, thus prompting such reports by consortia of panel users.

In the context of a lack of extensive analytical and clinical validation studies, this study may contribute to the surveillance of the assay performance specifically regarding *H. influenza*e. Among the *H. influenzae* false-positive results reported in the literature (Naccache et al., 2018; Boudet et al., 2019), it remains unclear whether the false-positive results are due to pre-analytic issues, sample contamination or to an analytical artifact. However, in our study, sample contamination seems unlikely given the rigorous handling of the samples, the analyses performed in two different laboratories, and the absence of frequent false-positive results for *Streptococcus pneumoniae* for instance, which could be at least as frequent as an external contaminant; qPCR results revealed to be all negative, thus suggesting, at least in the tested samples, that the positive results of the *H. influenzae* FilmArray^®^ ME Panel were likely false-positive. False-positive results highlight the precautions needed to perform rapid diagnostic tests to avoid cross-contamination from the environment and especially from the personnel in contact with the sample during the pre-analytical and analytical phases of testing.

Of note, there is also a lack of validation studies assessing the performance of the assay for each pathogen individually. Specifically regarding *H. influenzae*, the sensitivity of the FilmArray^®^ ME Panel compared to culture may in fact confer an advantage for CNS infection diagnosis, as reported in a recent study (Nestor et al., 2019). Nevertheless, our study raises the issue of its potential lack of specificity for *H. influenzae*, as reported by others (Naccache et al., 2018; Boudet et al., 2019). Globally, the conclusions drawn on the basis of the performances of the assay based on the few validation studies available are notably limited by the low number of samples tested for each pathogen.

Our study has some limitations. Specific qPCR was not systematically performed in the context of an insufficient available CSF sample. Nevertheless, qPCR results revealed to be all negative, thus suggesting, at least in the samples tested, that the positive results of the *H. influenzae* FilmArray^®^ ME Panel were likely false-positive. Some laboratory analyses were missing, precluding a rigorous classification of some cases. The retrospective review of clinical charts did not identify any antibiotic adverse events or *C. difficile* infections, although we cannot exclude information bias due to the lack of reporting or the occurrence of such events after hospitalization.

In conclusion, this study highlights the need for caution for *H. influenzae*-positive results with the FilmArray^®^ ME Panel. Meningoencephalitis syndromic panel results should always be interpreted together with clinical manifestations, CSF analysis, and other microbiological results. The use of a specific *H. influenzae* qPCR on CSF samples should be systematically considered as a confirmatory assay.

## Data Availability Statement

The original contributions presented in the study are included in the article/**Supplementary Material**. Further inquiries can be directed to the corresponding author.

## Ethics Statement

The studies involving human participants were reviewed and approved by the Geneva and Basel cantonal ethics commissions. Written informed consent for participation was not required for this study in accordance with the national legislation and the institutional requirements.

## Author Contributions

M-CZ, AE, and JS contributed to the conception of the work. M-CZ, VH, AC, and GR contributed to the data collection. M-CZ, AC, VH, DG, AE, and JS contributed to the data analysis and interpretation. M-CZ wrote the article. AE and JS contributed to the critical revision of the article. All authors contributed to the article and approved the submitted version.

## Conflict of Interest

The authors declare that the research was conducted in the absence of any commercial or financial relationships that could be construed as a potential conflict of interest.

## Supplementary Material

Supplementary Table 1 Detailed results of the BioFire FilmArray^®^ ME Panel for positive *H. influenzae* cerebrospinal fluid samples (n = 18).
