# Supplementary material for: Unexpectedly High False-Positive Rates for Haemophilus influenzae Using a Meningoencephalitis Syndromic PCR Panel in Two Tertiary Centers
Source: Front Cell Infect Microbiol. 2021 Mar 8;11:639658. doi: 10.3389/fcimb.2021.639658 (PMC7982903; doi:10.3389/fcimb.2021.639658)
Supplement: Supplementary Table 1 — Detailed results of the BioFire FilmArray® ME Panel for positive H. influenzae cerebrospinal fluid samples (n = 18). [file Table_1.docx]

Supplementary Material

# Supplementary Table

**Table S1:** Detailed results of the BioFire FilmArray® ME panel for positive *H. influenzae* cerebrospinal fluid samples (n=18).

| **Patient number** | **Initial BioFire FilmArray^®^ ME Panel test** | | | **Repeat BioFire FilmArray^®^ ME Panel test** | | |  |  |
| --- | --- | --- | --- | --- | --- | --- | --- | --- |
|  | Target 1  (number of  amplification curves) | Target 2  (number of  amplification curves) | Result for  *H. influenzae* | Target 1  (number of  amplification curves) | Target 2  (number of  amplification curves) | Result for  *H. influenzae* | | |
| 1 | POS (3) | NEG | Detected |  |  | ND | |  |
| 2 | POS (3) | NEG | Detected | POS (3) | NEG | Detected | |  |
| 3 | POS (3) | NEG | Detected |  |  | ND | |  |
| 4 | POS (3) | POS (3) | Detected | POS (2) | NEG | Detected | |  |
| 5 | POS (3) | NEG | Detected |  |  | ND | |  |
| 6 | NA | NA | Detected | NA | NA | Not detected * | |  |
| 7 | POS (3) | NEG | Detected |  |  | ND | |  |
| 8 | POS (3) | NEG | Detected |  |  | ND | |  |
| 9 | POS (3) | NEG | Detected |  |  | ND | |  |
| 10 | POS (3) | NEG | Detected |  |  | ND | |  |
| 11 | POS (3) | NEG | Detected |  |  | ND | |  |
| 12 | POS (3) | NEG | Detected |  |  | ND | |  |
| 13 | POS (3) | NEG | Detected |  |  | ND | |  |
| 14 | POS (2) | NEG | Detected |  |  | ND | |  |
| 15 | POS (3) | NEG | Detected |  |  | ND | |  |
| 16 | POS (3) | NEG | Detected |  |  | ND | |  |
| 17 | POS (3) | NEG | Detected |  |  | ND | |  |
| 18 | POS (2) | NEG | Detected | NEG | NEG | Not detected | |  |

* The BioFire FilmArray® ME Panel was not repeated on the CSF sample of this patient. The FilmArray^®^ Blood Culture Identification Panel was performed considering CSF direct examination revealing Gram positive cocci: the panel was negative for *H. influenzae* and positive for *Staphylococcus aureus*, and confirmed CSF direct examination and culture results. Importantly, this assay is not validated on CSF samples and negative *H. influenzae* results should be interpreted cautiously.

According to the manufacturer’s instructions, the detection of one target only does not suggest that the result is a false positive result.

*Abbreviations*: nb: number of amplification curves (maximum of 3) for each target; POS: positive; NEG: negative; ND: not done; NA: non-available data due to technical reasons
